# Self-Powered Photodetectors with High Stability Based on Se Paper/P3HT:Graphene Heterojunction

**DOI:** 10.3390/nano14231923

**Published:** 2024-11-29

**Authors:** Xuewei Yu, Yuxin Huang, Pengfan Li, Shiliang Feng, Xi Wan, Yanfeng Jiang, Pingping Yu

**Affiliations:** School of Integrated Circuits, Jiangnan University, Wuxi 214122, China; 6231916052@stu.jiangnan.edu.cn (X.Y.); 6231916005@stu.jiangnan.edu.cn (Y.H.); 6231916037@stu.jiangnan.edu.cn (P.L.); 6201924073@stu.jiangnan.edu.cn (S.F.); xwan@jiangnan.edu.cn (X.W.); jiangyf@jiangnan.edu.cn (Y.J.)

**Keywords:** self-powered photodetector, selenium, organic semiconductor materials, graphene, stability

## Abstract

Photodetectors based on selenium (Se) have attracted significant attention because of their outstanding optoelectronic characteristics, including their rapid reactivity and high photoconductivity. However, the poor responsivity of pure Se limits their further development. In this study, a novel Se-P/P3HT:G photodetector was designed and fabricated by combining an organic semiconductor made of poly-3-hexylthiophene mixed with graphene (P3HT:G) with self-supporting Se paper (Se-P) via spin-coating process. The device possesses a dark current of around 4.23 × 10^−12^ A and self-powered characteristics at 300–900 nm. At zero bias voltage and 548 nm illumination, the Se-P/P3HT:G photodetector demonstrates a maximum photocurrent of 1.35 × 10^−9^ A (745% higher than that of Se-P at 0.1 V), a quick response time (16.2/27.6 ms), an on/off ratio of 292, and a maximum detectivity and responsivity of 6.47 × 10^11^ Jones and 34 mA W^−1^, respectively. Moreover, Se-P/P3HT:G exhibits superior environmental stability. After one month, the photocurrent value of the Se-P/P3HT:G device held steady at 91.4% of its initial value, and even following pre-treatment at 140 °C, the on/off ratio still remained 17 (at a retention rate of about 5.9%). The excellent thermal stability, environmental reliability, and optoelectronic performance of this heterojunction structure offer a useful pathway for the future advancement of high-performance optoelectronic devices.

## 1. Introduction

Photodetectors capable of discerning diverse and intricate environmental data have attracted significant attention across a range of fields, including medicine, space exploration, military operations, and scientific research [1,2,3,4]. Self-powered photodetectors present a variety of promising application prospects because of their exceptional flexibility, portability, and no external energy consumption. However, to obtain high-performance photodetectors, there are several challenges that need to be solved, such as a limited light response range, poor long-term stability, and difficulties integrating and producing them at a large scale [5,6,7,8,9,10,11]. Photodetectors use the built-in electric potential of heterojunctions, or Schottky junctions, to quickly separate electron–hole pairs. Compared to Schottky junctions, heterojunctions are more suited to photodetectors because they do not require frequent adjustments of the height and stability of the Schottky barrier throughout the material and manufacturing processes [12]. Doping is one of the most common methods for improving their optoelectronic performance. However, issues including restrictions on the implementation of doping, selecting appropriate dopants, and modifying the doping ratios still need to be resolved. Therefore, it is highly beneficial to look for appropriate materials and an inexpensive, user-friendly process for making self-powered, high-performance photodetectors.

Selenium (Se) is a p-type semiconductor with a boiling point of 684.9 °C and a low melting point of 217 °C, which makes it suitable for preparing nanostructures with different morphologies at low temperatures using chemical vapor deposition (CVD). The current of Se can be changed rapidly with illumination at different light intensities, so it can be used widely for photoelectric detection in the ultraviolet–visible spectrum [13,14]. The Se microtube photodetector reported by Hu et al. exhibited a maximum responsivity of 19 mA W^−1^ at 610 nm with the range of 300 to 700 nm [15]. The low responsivity of elemental Se limits its application because of high optical reflectance. Heterojunction structures combining Se and other semiconductors (organic and inorganic) have been used to harness self-powered characteristics with high photodetection properties. Organic semiconductor materials provide various advantages, including tunable band gaps, high solubility, ease of processing, a light weight, flexibility, and the ability to reduce incident light reflection to enhance the light absorption rate [16,17,18]. Organic semiconductors (PPy, PANI, PEDOT, and Spiro-MeOTAD) have been deposited onto low-dimensional Se microtubes to create self-powered photodetectors, exhibiting high switch ratios of about 156–1300, a responsivity of 5.5–120 mA W^−1^, and fast response times in the order of milliseconds [19,20,21,22,23,24,25,26,27,28]. However, in the above organic semiconductors, disadvantages such as a low carrier mobility, poor stability, susceptibility to environmental effects, and difficulty in processing may exist. Therefore, it is essential to choose organic materials with a high stability and high carrier mobility to improve their self-powered photodetection performance.

Poly(3-hexylthiophene) (P3HT) is the most researched polythiophene derivative, which is utilized in solar cells [29], thermoelectric devices [30], and photodetectors [31] because of its outstanding stability, broad bandgap, excellent hole transport capability, superior solubility, and processability. In the self-biased mode, ZnO/Pt/P3HT hybrid ultraviolet (UV) photodiodes exhibit a specific detectivity of 2 × 10^12^ Jones, photosensitivity of 46, and photoresponsivity of 14.8 mA W^−1^, respectively. These devices exhibit quick response times of only 45 ms, strong UV sensitivity, and low power consumption [32]. P3HT has superior adhesion and stability. Adding P3HT to a ZnO/CsSnBr3 heterojunction photodetector improved its specific detectivity to 1.40 × 10^14^ Jones and its responsivity to 1.56 A W^−1^ in self-driven mode and reduces the dark current due to the surface passivation effect of P3HT on CsSnBr_3_ [33]. Graphene exhibits a high carrier mobility and absorptivity. A Si:Ag/graphene infrared photodetector exhibited external quantum efficiency (EQE) values of 97.26% and 7.37% at 1310 nm and 1550 nm, respectively. This outstanding photoelectric performance is due to the high optical absorption of defects at a deep level caused by highly doped Ag impurities and the high mobility of graphene, which can effectively generate a photocurrent [34]. Graphene can improve the photoelectric performances of WO_3_ devices due to its ability to both absorb visible light and transfer photogenerated electrons. Compared to pure WO_3_ devices, the responsivity (0.253 A W^−1^) and specific detectivity (5.136 × 10^11^ Jones) of WO_3_/graphene increased by 53% and 52%, respectively [35]. Adding graphene to P3HT at a 50% ratio results in an expanded absorption spectrum in the UV–visible region and up to a 34% increase in the absorption area, attributed to the ability of graphene to capture light [36]. It is also possible to obtain greater conductivity and reduce the tangent losses [37]. A phototransistor fabricated by combining graphene with P3HT demonstrated a high hole mobility of up to 18 cm^2^ V^−1^ s^−1^, a responsivity of 18 A W^−1^ in the visible light range, and a short rise/fall response time of 2.2/2.3 ms, which was explained by the fast charge transfer from the P3HT to the graphene [38]. Therefore, P3HT:graphene (P3HT:G) may provide a promising pathway for the current photodetection industry.

**Figure 1:**
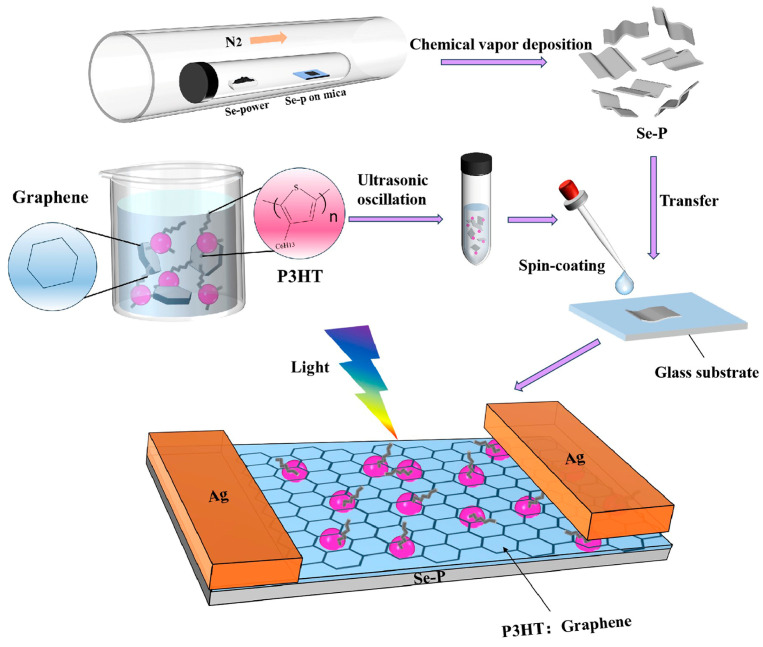
Schematic illustration of the fabrication process for Se-P/P3HT:G heterojunction photodetectors.

In this work, self-supporting Se paper (Se-P) was prepared using the vapor deposition approach. Se-P/P3HT and Se-P/P3HT:G photodetectors were fabricated using spin- coating process as shown in Figure 1. P3HT:G was utilized to improve the mobility, light absorption, and generation of additional photogenerated carriers. In comparison to pure Se-P, the responsivity of Se-P/P3HT and Se-P/P3HT:G increased 2.7 times (from 8.68 mA W^−1^ to 23.6 mA W^−1^) and 3.9 times (from 8.68 mA W^−1^ to 34 mA W^−1^) over at 548 nm. The photoelectric characteristics of these two kinds of devices at zero bias and the corresponding physical mechanisms were systematically analyzed, with the corresponding energy band diagrams.

## 2. Materials and Methods

### 2.1. Preparation of Se-P

A ceramic boat filled with Se powder (A.R. 99%) was placed in the center of a tubular furnace, with high-purity nitrogen injected 2–3 times to remove the air from the furnace. A quartz tube was kept in a low-pressure environment before heating. Then, the temperature of the center of the tube furnace was boosted by 10 °C per minute to 260 °C, and this was held at 260 °C for 90 min before allowing the tube furnace to cool naturally to room temperature. A layer of Se was obtained on the inner wall of the quartz tube and the mica substrate at the end of the tube after adding new Se powder and repeating the above experimental steps four times. In the following step, the mica substrate or the quartz tube with the Se film was dried for two hours at 90 °C in a blast-drying oven. After that, the Se film was collected from the mica substrate or the tube wall at room temperature using tweezers.

### 2.2. Preparation of P3HT:G

Graphene nanosheets were dispersed in dimethyl sulfoxide (1 mg mL^−1^), isopropanol, and ethylene glycol mixed solutions and ultrasonicated for 24 h at 5 °C. A total of 25 μL of the graphene dispersion solution was added to 1 mL of P3HT solution and stirred for 12 h at room temperature to obtain a P3HT:G (at a weight ratio of about 1:0.025) mixed solution.

### 2.3. Preparation of Se-P/P3HT:G Heterojunctions

The Se-P (0.5 cm × 0.5 cm) was transferred onto a glass substrate. P3HT:G mixed solution was deposited onto the Se-P piece using the spin coating method at 1000 rpm for 30 s. Finally, Ag paste was deposited onto the Se-P and P3HT:G as their electrodes, respectively, followed by 10 min of thermal annealing in air at 50 °C.

### 2.4. Material Characterization

Samples were examined using a JSM-7000F (Japan Electronics Co., Ltd., Tokyo, Japan) scanning electron microscope (SEM) to determine their morphology and structure. A Bruker D8-A25X (Bruker Scientific Technology Co., Ltd., Karlsruhe, Germany) X-ray diffractometer (XRD) was used to obtain the X-ray diffraction patterns. A Varian Cary 500 UV-vis spectrophotometer (Agilent Technology Co., Ltd., Santa Clara, CA, USA) was used to measure the UV–visible absorption spectra, and a LabRam-1B Raman spectrometer was used to acquire the Raman spectra at an excitation wavelength of 632.8 nm. The optoelectronic properties of the heterojunctions were investigated using Keithley 4200-based semiconductor (Teck Technology Co., Ltd., Thousand Oaks, CA, USA) testing equipment at room temperature.

## 3. Results and Discussions

**Figure 2:**
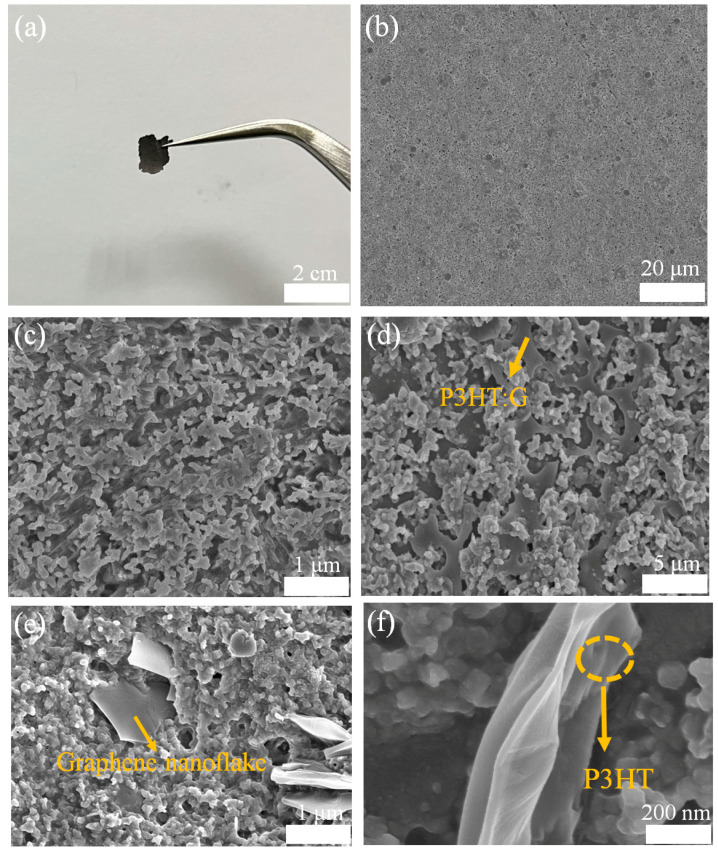
(**a**) Photograph of Se-P, and SEM images of Se-P at low (**b**) and high (**c**) magnification. A SEM image of Se-P/P3HT:G (**d**), SEM images of a graphene nanoflake (**e**), and P3HT (**f**) in the Se-P/P3HT:G heterojunction.

Good self-supporting Se-P was prepared using the CVD method, as seen in Figure 2a, with an area of about 1 cm^2^, which could readily be transferred onto the substrate. The Se-P exhibited a uniform, multilayer, porous surface (Figure 2b) formed of Se nanoribbons with lengths ranging from 3 to 8 μm (Figure 2c). This porous multilayer structure of the Se nanoribbons is a great carrier for depositing organic semiconductors. The P3HT:G mixed solution successfully attached to the surface of the Se-P (Figure 2d), which reduced the carrier transport distances. Graphene nanoflakes are clearly visible in Figure 2e, with their diameter and thickness varying from 300 nm to 1 μm and around 50 nm, respectively. The P3HT solution adhering to the graphene nanosheets is clearly embedded into the surface of the Se-P, as seen in the high-magnification SEM image in Figure 2f.

**Figure 3:**
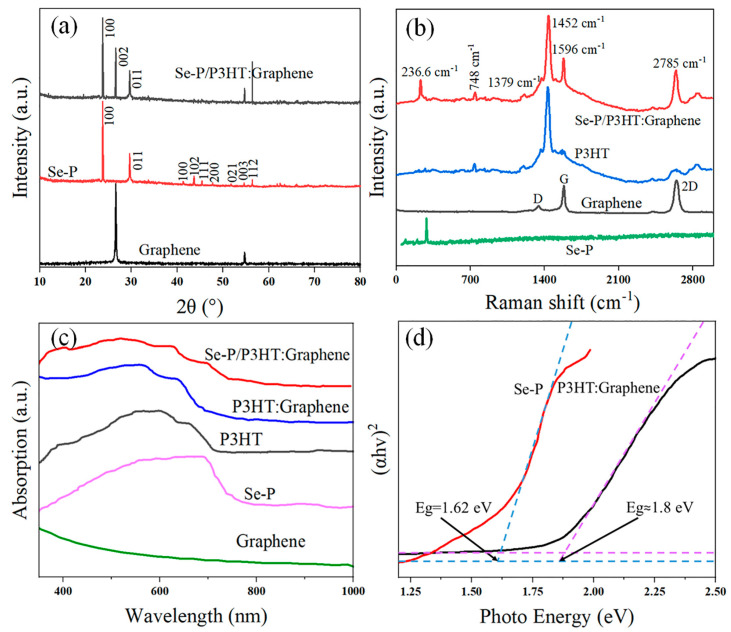
(**a**) XRD patterns of Se-P, graphene, and Se-P/P3HT:G; (**b**) Raman spectra of Se-P, Graphene, P3HT, and Se-P/P3HT:G; (**c**) UV-vis absorption spectra of P3HT, graphene, P3HT:G, Se-P, and Se-P/P3HT:G; and (**d**) Eg of Se-P and P3HT:G.

The XRD patterns of Se-P, graphene, and Se-P/P3HT:G are shown in Figure 3a. Se-P has high crystallinity and belongs to trigonal selenium (t-Se), which matches the standard PDF card JCPDS No. 65-1876. Peaks characteristic of graphene are located at 26.4° and 54.7°, respectively, which are consistent with the crystal faces of the (002) and (004) crystal planes. The P3HT:G layer reduces the intensity of the Se-P diffraction peaks beyond 40°. The primary diffraction peaks at the (100) and (011) crystal planes show no change in intensity, suggesting that the addition of P3HT:G has a slight influence on the crystallinity of Se-P. The Raman spectra of Se-P, graphene, P3HT, and Se-P/P3HT:G are shown in Figure 3b. The D-band (1340 cm^−1^, disordered C-C structure), 2D-band (2685 cm^−1^, double-resonant) and conventional G-band (1594 cm^−1^, sp2-hybridized carbon) of graphene are present. The number of layers and defect content of graphene can be calculated via the G/2D and D/G intensity ratios. The I_G/2D_ and I_D/G_ of graphene are approximately 0.61 and 0.1, respectively, indicating the presence of monolayer graphene [39]. P3HT contains the C-H stretching vibration at 2896 cm^−1^ [40], the C-C intra-ring stretch mode at 1379 cm^−1^, the symmetric C=C stretch mode at 1440 cm^−1^, the inter-ring C-C stretch at 1208 cm^−1^, and the C-S stretching vibration at 748 cm^−1^ [41]. The Raman spectrum of Se-P/P3HT:G shows the G and 2D bands of P3HT:G at 1596 and 2785 cm^−1^, respectively, since the C-C stretching vibration of P3HT suppresses the D peak. In addition, the G band shifts from 1593 cm^−1^ to 1596 cm^−1^, which also indicates that charge transfer has occurred between the P3HT and graphene [42]. The characteristic peak at 236.6 cm^−1^ corresponds to the Se-P bond. These findings indicate the successful fabrication of a Se-P/P3HT:G heterojunction structure.

The UV-vis absorption spectra of P3HT, graphene, P3HT:G, Se-P, and Se-P/P3HT:G are shown in Figure 3c. P3HT shows a wide range of absorption from 400 to 700 nm, culminating at about 600 nm, which corresponds to the π-π* transition of the P3HT conjugated system [43]. After adding graphene to P3HT, the P3HT chains unfold at the edges and flaws of the graphene surface [44]. The absorption peak of P3HT:G is shifted towards the infrared region, broadening the absorption spectrum, with a noticeable increase in the absorbance between 350 nm and 550 nm [45]. The absorption cutoff edge of Se-P/P3HT:G spreads from 670 nm (equal to P3HT:G) to 724 nm due to the capacity of Se-P to broaden the response range. The UV-vis absorption spectra depicted in Figure 3c can be used to calculate the bandgaps of the P3HT:G and Se-P shown in Figure 3d, which are 1.8 eV and 1.62 eV, respectively.

**Figure 4:**
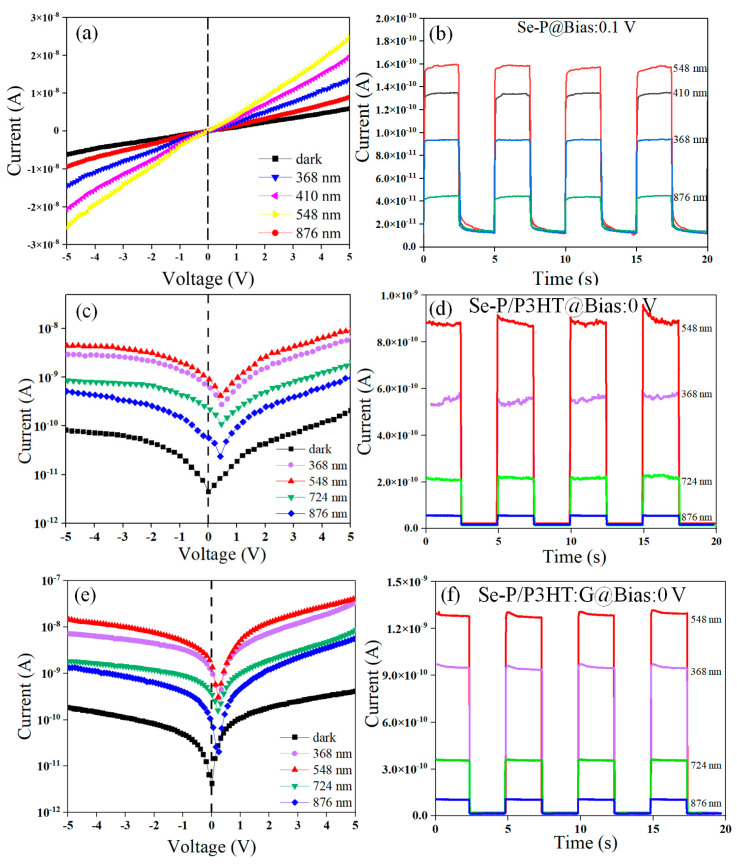
Se-P I-V curves (**a**) and I-t curves (**b**) at various wavelengths; Se-P/P3HT I-V curves (**c**) and I-t curves (**d**) at various wavelengths; and Se-P/P3HT:G I-V curves (**e**) and I-t curves (**f**) at various wavelengths.

The current–voltage (I-V) curves of Se-P under darkness (0.10 mW cm^−2^) and illumination at 368 nm (0.52 mW cm^−2^), 410 nm (0.54 mW cm^−2^), 548 nm (1.31 mW cm^−2^), and 876 nm (0.32 mW cm^−2^) are shown in Figure 4a. Se-P exhibits the lowest current in darkness. The light current (I_light_) of Se-P gradually rises in the ultraviolet region (368 nm), reaches its maximum in the visible region (548 nm), and then drops to 10 nA at 876 nm under a 5 V bias, indicating good broadband photoelectronic performance. The I–V curves of Se-P at forward and reverse bias are both linear, indicating ohmic contact between the Ag electrode and the Se-P. The current–time (I-t) curves of Se-P under a 0.1 V bias at various wavelengths (Figure 4b) illustrate its good photoelectric properties. At 368 nm, 410 nm, 548 nm, and 876 nm, the photocurrent of Se-P is 93 pA, 134 pA, 160 pA, and 44 pA, respectively, while the dark current is roughly 12 pA, and the on/off (I_light_/I_dark_) ratios can be calculated to be 7.8, 11.1, 13.3, and 3.7, respectively.

The Se-P/P3HT heterojunction formed of P3HT deposited onto the surface of Se-P exhibits a dark current of approximately 4.37 × 10^−12^ A (Figure 4c) and an open-circuit voltage (V_oc_) value of 0.18 V. A built-in electric field is created that enables a photoresponse without the need for an external voltage, demonstrating the self-powered capability of the device. The I-V curves of the Se-P/P3HT photodetector in logarithmic coordinates present an asymmetric pattern. Although the I_light_ values under a positive and negative bias voltage are different, the rectification ratio is relatively small, at about 2, which may be caused by the low barrier height of the ohmic contact and the different degrees of contact of the Ag electrode. The I-t curves of the Se-P/P3HT device in Figure 4d show the maximal photocurrent of 8.89 × 10^−10^ A at 548 nm light irradiation, with an on/off ratio of 203. Under 876 nm light irradiation, the photocurrent decreases to 7.63 × 10^−11^ A, with an on/off ratio of 17 due to the weak absorption beyond 700 nm. The Se-P/P3HT:G photodetector exhibits a decreased dark current of 4.23 × 10^−12^ A due to the high resistance of graphene and a built-in potential (V_oc_) of 0.2 V under illumination, demonstrating its self-powered capability (Figure 4e).The I_light_ value of the Se-P/P3HT:G device at 548 nm and a 0 V bias is 1.36 × 10^−9^ A, with the on/off ratio increased to 292, implying the addition of graphene improves the carrier transport capability inside the P3HT:G active layer, leading to a rise in the photocurrent values (Figure 4f) [46]. The bigger built-in electric field the separation of electron–hole pairs more efficiently promotes, considerably increasing the photocurrent and decreasing the response time.

**Figure 5:**
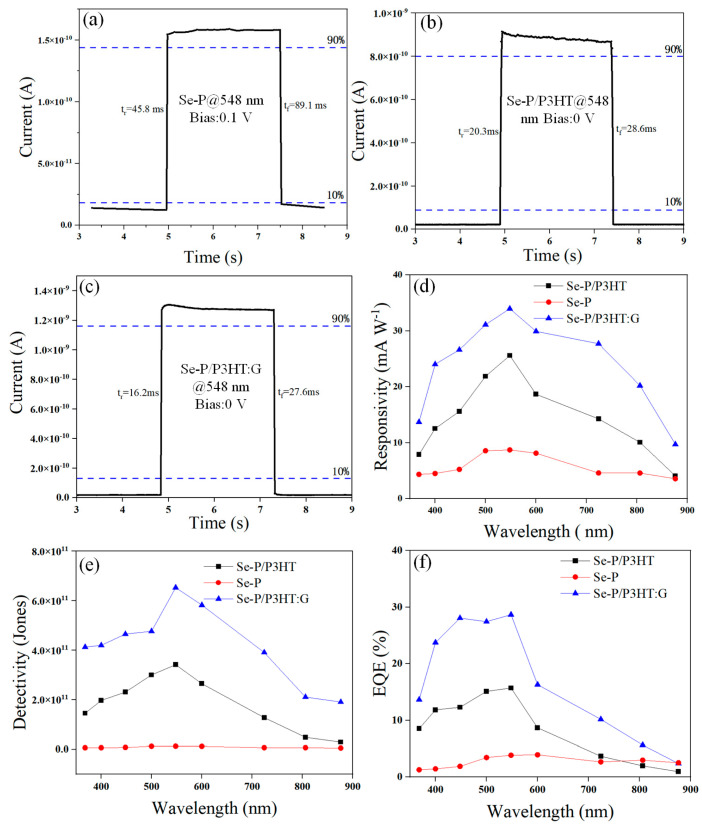
Rise/fall times of the Se-P at 548 nm (1.31 mW cm^−2^) under 0.1 V (**a**) and Se-P/P3HT (**b**) and Se-P/P3HT:G (**c**) at 548 nm and 0 V bias in a single cycle. Responsivity (**d**), specific detectivity (**e**), and external quantum efficiency (**f**) of Se-P, Se-P/P3HT, and Se-P/P3HT:G versus the wavelength range of 348–876 nm at 0 V bias.

One of the most important features of any photodetector is its rise/fall time. Under illumination, the time it takes for a photodetector to climb from 10% to 90% of its saturated photocurrent is called the rise time, and the time it takes for it to drop from 90% of its saturated photocurrent to 10% is called the fall time. The rise and fall times of the Se-P (Figure 5a), Se-P/P3HT (Figure 5b), and Se-P/P3HT:G (Figure 5c) devices during one cycle are 45.8 ms/89.1 ms, 20.3 ms/28.6 ms, and 16.2 ms/27.6 ms under 548 nm illumination at zero bias, respectively. Because of the π–π stacking of graphene at various angles in the P3HT:G mixed layer, which favors the establishment of a continuous pathway for charge transport to increase the recombination rate of electrons and holes, the rise/fall time of the Se-P/P3HT:G device is 125%/104% faster compared to that of the Se-P/P3HT device. Furthermore, both the process of the drop in the current of carrier recombination under dark conditions and the process of an increase in current in the device’s response under illumination are linear.

To determine the optoelectronic capability of the devices, the key parameters responsivity (R), specific detectivity (D*), and external quantum efficiency (EQE) of the Se-P, Se-P/P3HT, and Se-P/P3HT:graphene are evaluated using the equations below:

(1) $$R=\frac{I_{ph}}{PS}$$

(2) $$D*=\frac{R}{{\left(2qI_{d}/S\right)}^{\frac{1}{2}}}$$

(3) $$EQE=\frac{Rq\lambda }{hc}$$

where I_ph_ is the photocurrent, defined as I_light_ − I_d_; I_d_ is the dark current; P is the power density of incident light; S is the effective irradiated area of the devices; λ is the light wavelength; c is the speed of light; h is the Planck constant; and q is the electron charge.

The calculated R values for the Se-P, Se-P/P3HT, and Se-P/P3HT:G devices at 0 V bias increase and subsequently decrease as a function of wavelengths, as displayed in Figure 5d. The maximal responsivity values for Se-P/P3HT and Se-P/P3HT:G are 23.6 mA W^−1^ and 34 mA W^−1^, generated at 548 nm, which are higher than that of Se-P, at 8.68 mA W^−1^, respectively. Compared to the Se-P/P3HT (3.41 × 10^11^ Jones) device and the Se-P device (1.46 × 10^10^ Jones), the D* values of Se-P/P3HT:G, as shown in Figure 5e, are superior between 348 and 876 nm in wavelength at 0 V bias, with a maximum value of 6.47 × 10^11^ Jones at 548 nm, indicating its higher capability to detect optical signals under the influence of fixed noise. At 548 nm, the highest EQE values of the Se-P/P3HT and Se-P devices are 15.2% and 3.94%, which are 67.2% and 17.4% lower than those of the Se-P/P3HT:G device (22.6%), respectively. The built-in electric field quickly separates the numerous electron–hole pairs produced by the light absorption of P3HT:G, resulting in the maximum spectral responsiveness of Se-P/P3HT:G occurring at 548 nm. Nevertheless, because of the decreasing penetration depth of photon light and the recombination of the electron–hole carriers produced, the responsivity tends to decrease as the photoenergy increases beyond 548 nm. The Se-P/P3HT:G photodetector performs better in key parameters like R, D*, and EQE and displays a larger photocurrent, a greater on/off ratio, and a faster response speed when compared to Se-P/P3HT and Se-P. The Se-P/P3HT:G photodetector exhibits a relatively high specific detectivity and performs similarly to other photodetectors that have been previously described using Se micro/nanostructures or the organic materials P3HT and graphene, as listed in Table 1.

**Figure 6:**
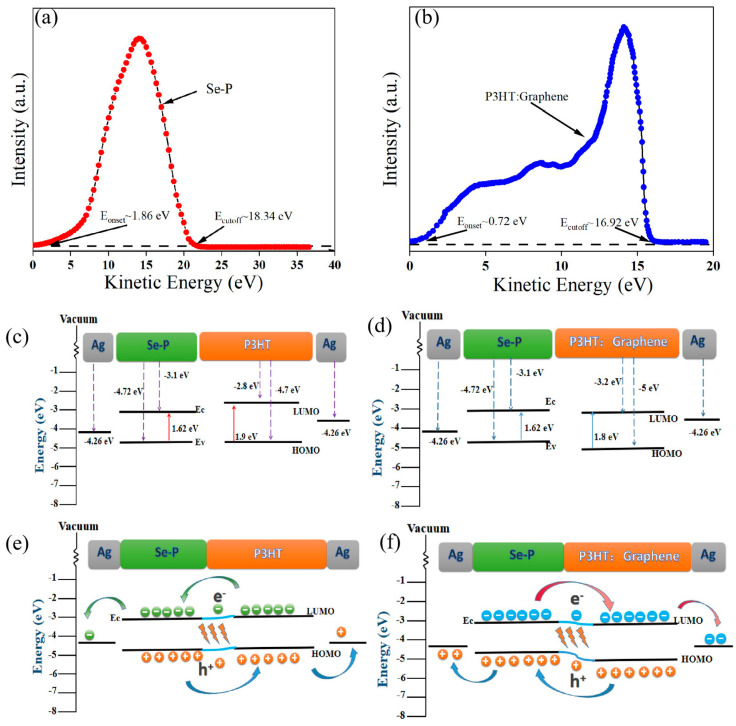
UPS spectra of Se-P (**a**,**b**) P3HT:G. Energy band diagrams of Se-P/P3HT (**c**) and Se-P/P3HT:G (**d**) before contact and without light illumination. Energy band diagrams of Se-P/P3HT (**e**) and Se-P/P3HT:G (**f**) after contact under light illumination.

The UPS spectra of Se-P (a) and (b) P3HT: G are shown in Figure 6. Based on the formula E_v_ = −[21.20 − (E_cutoff_ − E_onset_)], the valence band (E_v_) of Se-P and the highest occupied molecular orbital (HOMO) of P3HT:G are calculated as −4.72 and −5 eV, respectively. According to the E_g_ of Se-P (1.62 eV) and P3HT:G (1.8 eV), which is consistent with the cutoff wavelength calculated from the UV-vis absorption spectra (E_g_ = 1240/λ, λ is the wavelength of light), the E_c_ of Se-P (3.1 eV) and the lowest occupied molecular orbital (LOMO) of P3HT:G (3.2 eV) can be calculated, resulting in a type-II heterojunction with Ag as the electron and hole transport electrodes. The E_g_ of P3HT is 1.9 eV, with an E_c_ of 2.8 eV and an E_v_ of 4.7 eV (Figure 6c). The energy band difference in Se-P and P3HT is ∆E_c_ = 0.30 eV and ∆E_v_ = 0.02 eV. The discrepancies in the valence and conduction bands of Se-P and P3HT:G are ∆E_v_ = 0.28 eV and ∆E_c_ = 0.1 eV, respectively, as depicted in Figure 6d. When the contact surface is balanced, a built-in electric field is created as a result of the majority of the carriers’ diffusion. The photogenerated carriers are separated by this built-in electric field, and the drift motion creates a continuous photocurrent. Under light, electrons transition from P3HT to Se-p, and holes migrate upward from Se-p to P3HT, forming a current at the electrode (Figure 6e). When light illuminates the heterojunction, the electrons in the Se-P and P3HT:G valence bands absorb the light energy, moving to the conduction band and leaving holes in the valence band. A continuous current is created when the photogenerated electrons migrate from the Se-P conduction band to the P3HT:G conduction band, and the holes migrate from the P3HT:G valence band to the Se-P valence band owing to the energy disparities between the conduction and valence bands on both sides. With a work function of about 4.5 eV [51], which lies between the HOMO (highest occupied molecular orbital) and the LUMO (lowest unoccupied molecular orbital) of the P3HT:G, graphene can function as an electron acceptor, combining with the electron donor P3HT to form a continuous electron pathway, which can enhance the conductivity with a negligible effect on the bandgap. When graphene is added to P3HT, the energy discrepancy between the two materials facilitates the transfer of holes from graphene to P3HT, creating a built-in field that equilibrates the Fermi level and causes the LUMO energy levels to drop and the HOMO energy levels to rise. Proper band alignment between graphene and P3HT significantly improves the photoresponse. Compared to the Se-P/P3HT photodetector, the Se-P/P3HT:G device exhibits self-powered properties, a higher photocurrent, and a faster response speed.

**Figure 7:**
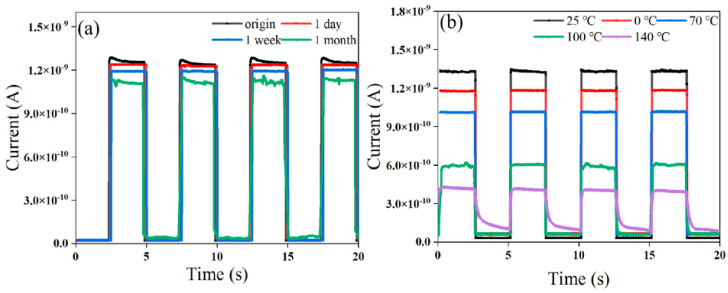
At 548 nm and 0 V bias, I-t curves (**a**) of Se-P/P3HT:G device left for 1 day, 1 week, and 1 month and I-t curves (**b**) of Se-P/P3HT:G device after treatment at 0 °C, 25 °C, 70 °C, 100 °C, and 140 °C, respectively.

In organic-based electronics, environmental stability is still a major problem, even with high performance. To further investigate the performance, the I-t curves of the unpackaged Se-P/P3HT:G device when placed at room temperature for one day, one week, and one month at 548 nm under 0 V bias are depicted in Figure 7a. After one month, the I_light_ (1.24 × 10^−9^ A) of the Se-P/P3HT:G photodetector still remains 91.4% of the initial photocurrent (1.36 × 10^−9^ A), suggesting that the device has excellent environmental stability and very little long-term performance degradation. However, if the device is left on for a long, extended period, I_light_ becomes unstable and exhibits significant oscillations and current spikes. As shown in Figure 7b, the Se-P/P3HT:G device displays the highest I_light_ (1.36 × 10^−9^ A) and the minimum I_d_ (4.23 × 10^−12^ A) with an on/off ratio of around 292 under 548 nm illumination at zero bias and 25 °C. After pre-treatment at 0 °C, the concentration and mobility of the charge carriers of the Se-P/P3HT:G device are dropped, leading to a lower photocurrent and dark current. Because of the lower hole transport capabilities of P3HT beyond 25 °C, the reverse cutoff properties of the device (i.e., carriers may more readily traverse the barrier zone) are weakened as the temperature increases, resulting in the I_light_ falling and the I_d_ increasing. After pre-treatment at 140 °C, the I_light_ (4.39 × 10^−10^ A) remains 32.2% of the original value at 25 °C, while the I_d_ increases to around 2.57 × 10^−11^ A, with an on/off ratio of 17, but the response time extends to 214 ms/1.84 s due to the increase in the electron–hole recombination rate and the decrease in the carrier lifetime. Selecting suitable organic semiconductor materials at the optimal processing temperature for preparing photoelectric devices will be a potential method to solve the problem of photoelectric performance being affected by temperature changes in future work.

## 4. Conclusions

In conclusion, a novel Se-P/P3HT:G broadband photodetector was successfully fabricated with good self-powered properties and high environmental stability by depositing P3HT:G mixed solution onto Se-P using the spinning method. With a high on/off ratio of 292, exceptional responsivity (34 mA W^−1^), a great specific detectivity of 6.47 × 10^11^ Jones, and a quick response time (16.2 ms/27.6 ms) at 548 nm, the Se-P/P3HT:G photodetector has a distinguished performance under 0 V bias. In comparison to the Se-P/P3HT photodetector, the Se-P/P3HT:G photodetector exhibits a 144% increase in its responsivity and a two-fold enhancement in its specific detectivity under 0 V bias at 548 nm, showing that the addition of graphene can significantly increase the conductivity and built-in potential of the device. With a millisecond-level response time, the response speed satisfies the needs of the majority of applications. Therefore, this work presents a novel method for achieving a simple, inexpensive, stable photodetector, which may provide a new path for photodetectors in the future.

## Figures and Tables

**Table 1:** Comparison of the performance of Se-P/P3HT:G, Se-P/P3HT, and other heterojunction photodetectors at 0 V bias.

Photodetectors	Light (nm)	R (mA W^−1^)	D* (Jones)	On/Off Ratio	Rise/Fall Time	Refs.
Se-P/P3HT:G	300–725	34	6.47 × 10^11^	292	16.2 ms/27.6 ms	This work
Se-P/P3HT	300–725	23.6	3.41 × 10^11^	203	45.8 ms/89.1 ms	This work
Se/PEDOT	300–700	5.5	1.76 × 10^10^	50	1.00 ms/9.78 ms	[28]
Se/Spiro-MeOTAD	350–800	36.5		156	22 ms/35 ms	[19]
ZnO/Pt/P3HT	250–700	14.8	1.2 × 10^12^	1700	45 ms/45 ms	[32]
P3HT/ZnO NW		365	125	3.7 × 10^7^	0.1 s/0.1 s	[47]
InSe/Se	300–700	32	1.7 × 10^11^	500	30 ms/37 ms	[48]
TiO_2_/P3HT	300–700	0.037	1.63 × 10^10^	16.8	0.72 s/0.5 s	[49]
Au/TiO_2_/P3HT	300–700	0.25	2.9 × 10^10^	6.5	0.48 s/2.12 s	[49]
SnS_2_/Graphene	380–780	6.98	1 × 10^10^		4.53 s/4.53 s	[50]

## Data Availability

The data are contained within the article.
